# Reduced stomatal density in wheat overexpressing EPIDERMAL PATTERNING FACTOR1 differentially affects red and blue light responses

**DOI:** 10.1093/plphys/kiaf379

**Published:** 2025-09-05

**Authors:** Mengjie Fan, Delfi Dorussen, Hussein Gherli, Tracy Lawson

**Affiliations:** School of Life Sciences, University of Essex, Colchester CO4 3SQ, UK; School of Life Sciences, University of Essex, Colchester CO4 3SQ, UK; School of Life Sciences, University of Essex, Colchester CO4 3SQ, UK; School of Life Sciences, University of Essex, Colchester CO4 3SQ, UK

## Abstract

Stomatal pores govern the trade-off between CO_2_ assimilation and water loss, and optimizing their performance is critical for crop resilience, particularly under dynamic field environments. Here, we show that overexpression of *Triticum aestivum* EPIDERMAL PATTERNING FACTOR1 (TaEPF1) in bread wheat (*T. aestivum*) reduces leaf stomatal density (SD) in a leaf surface-specific manner, with a greater decline on the abaxial surface than on the adaxial surface. TaEPF1 *overexpressors* exhibited substantially lower stomatal conductance than wild-type (WT) control plants, which resulted in diffusional constraints that limited photosynthesis when measured under monochromatic red light. However, upon partial substitution of red light with blue light, EPIDERMAL PATTERNING FACTOR1 *overexpressors* displayed an amplified and rapid stomatal opening response, particularly on the abaxial surface, where relative conductance increased by up to 90% versus 49% observed in the WT. Despite anatomical limitations in maximum conductance rate, this blue light sensitivity effectively compensated for the lower baseline gas exchange. The enhanced sensitivity to blue light was also concomitant with lower intercellular CO_2_ levels under red light. When *g*_sw_ responses were normalized relative to SD, stomatal sensitivity to red light was reduced at the pore level, while blue light sensitivity increased, which was particularly evident during abaxial surface illumination. Finally, the transgenic lines maintained a 15% to 20% higher water use efficiency across light regimes. These findings show a compensatory mechanism in which genetically induced reduction in SD is offset by heightened blue light sensitivity.

## Teaser

EPIDERMAL PATTERNING FACTOR1 overexpression in wheat affects abaxial and adaxial stomatal density differently, and stomatal responses to blue light differentially compensate for reduced conductance.

## Introduction

Stomatal pores regulate the exchange of CO_2_ and water vapor between the plant interior and the atmosphere, thus playing a critical role in balancing photosynthetic carbon gain and water loss (Caine et al. 2019; Lawson and Matthews 2020). Guard cells surrounding these pores exhibit rapid, dynamic responses to many environmental stimuli, including light intensity, light spectra, vapor pressure deficit (VPD), and internal CO_2_ concentration (*C_i_*), which are essential for optimizing photosynthetic efficiency and maintaining water use efficiency (WUE) (Drake et al. 2013; Lawson and Vialet-Chabrand 2019). In natural fluctuating environments, the speed and magnitude of stomatal adjustments are particularly important for sustaining photosynthesis while mitigating water loss under transient light conditions (Kaiser et al. 2015; Buckley 2019; Lawson and Vialet-Chabrand 2019). Rapid opening to meet photosynthetic demands for CO_2_ improves carbon assimilation (*A*), while rapid closure under conditions when *A* has decreased (e.g. low light intensity) improves intrinsic WUE (*W_i_*) (Faralli et al. 2019; Papanatsiou et al. 2019; Battle et al. 2024).

Light quality, as well as intensity, plays a pivotal role in modulating stomatal behavior. These light responses have been separated into the red or mesophyll responses and the specific blue light response (Matthews et al. 2020). Traditionally, red light has been linked directly with photosynthetic activity and has been associated with lowering intercellular CO_2_ (*C_i_*) through enhanced CO_2_ uptake, which in turn promotes stomatal opening (Lawson et al. 2014; Ando and Kinoshita 2018; Taylor et al. 2024). There is considerable evidence to suggest that *C_i_* is not the only signal that mediates the red light responses and that *C_i_*-independent mechanisms exist (Messinger et al. 2006; Lawson 2009; Matrosova et al. 2015), although the exact mechanisms have not been fully elucidated (Mott et al. 2008; Lawson et al. 2018; Taylor et al. 2024). In contrast, blue light elicits a distinct and guard cell-specific response that operates effectively at low intensities (<50 µmol m^−2^ s^−1^) (Vialet-Chabrand et al. 2021), is not linked to photosynthesis and is reported to be up to 20 times more effective at inducing stomatal opening than red light (Doi et al. 2015; Hiyama et al. 2017; Inoue and Kinoshita 2017; Matthews et al. 2020). This blue light response is perceived by phototropins (PHOT1 and PHOT2) in the guard cells, which activate a signal transduction cascade involving the plasma membrane H⁺-ATPase, ion fluxes, and guard cell starch degradation to facilitate rapid stomatal opening (Inoue et al. 2008; Horrer et al. 2016; Inoue and Kinoshita 2017; Flütsch et al. 2020). The stomatal blue-light response can also aid leaf cooling under fluctuating light, which is critical to prevent heat stress (Zeiger and Field 1982; Schwartz and Zeiger 1984; Pearcy 1990; Lawson and Blatt 2014). Such dual regulation highlights that stomatal dynamics are governed not only by mesophyll-driven signals but also by direct light-signaling pathways that can adjust aperture independently of photosynthetic rates (Lawson 2009). Notably, Taylor et al. (2024) observed suppression of *C_i_-*independent stomatal opening under high *C_i_* conditions.

Improving WUE by manipulating stomatal density (SD) using genetic approaches has emerged as a promising strategy, which is becoming increasingly desirable under conditions of water scarcity (Doheny-Adams et al. 2012; Franks et al. 2015; Hughes et al. 2017; Hofmann et al. 2025). Overexpression of EPIDERMAL PATTERNING FACTOR1 (EPF1), which acts as a negative regulator of stomatal development by inhibiting the formation of stomatal precursors, has been shown to decrease SD and consequently, modulate gas exchange and photosynthetic performance (Hara et al. 2007; Hepworth et al. 2015; Dunn et al. 2019; Lu et al. 2019). Another emerging yet underexplored aspect of SD and the regulation of gaseous exchange is the differential patterning and behavior in adaxial (upper) and abaxial (lower) leaf surfaces (Driscoll et al. 2006; Wang et al. 2008; Wall et al. 2022; Driesen et al. 2023; Fan et al. 2025). Typically, the adaxial surface is exposed to higher light intensities, while the abaxial surface experiences more shade, leading to inherent differences in SD *and* responsiveness. Unlike many dicots that show higher SD on the abaxial surface, wheat exhibits amphistomatous leaves with relatively even distributions or even greater SD on the adaxial surface, suggesting a distinct adaptation pattern that may influence gas exchange dynamics (Driscoll et al. 2006; Wang et al. 2008; Zhao et al. 2020; Wall et al. 2022). Although these polarity cues and the environmental impact on these developmental pathways have been characterized in some species (Turner 1970; Knecht and O'Leary 1972; Wang et al. 2008; Sun et al. 2021), there is limited study on the similar mechanisms that operate in important monocots such as wheat. Differential regulation of stomatal development on these surfaces may have implications for gas exchange, especially when anatomical constraints, such as those with genetically reduced SD, infer limitations on CO_2_ uptake for photosynthesis. For example, in wheat the majority of gas exchange takes place via the adaxial surface (Wall et al. 2022), while the abaxial surface has often been considered the major contributor to leaf gas exchange in other species (Fanourakis et al. 2015; Richardson et al. 2017; Mochizuki et al. 2019), often due to reduced evaporative demand from this surface as it is not in direct sunlight. Wall et al. (2022) also demonstrated that stomatal behavior on one surface could not compensate for changes on the opposite side, suggesting independent functioning. The 2 surfaces (of wheat flag leaves) can receive direct illumination when growing erect, while when more horizontal, the 2 surfaces not only receive different light intensities but also different spectra due to absorption of specific wavelengths by the mesophyll (Day et al. 1994; Johnson et al. 2005; Brodersen and Vogelmann 2010; Théroux-Rancourt and Gilbert 2017), which has been shown to influence stomatal behavior (Wang et al. 2016).

Despite the well-established roles of red and blue light in driving stomatal dynamics, a critical gap remains in our understanding of how these responses may differ between the 2 surfaces and how engineered for reduced SD may influence these responses. In particular, it remained underexplored how the adaxial and abaxial leaf surfaces respond to direct illumination and whether compensatory physiological mechanisms exist that might offset the diminished anatomical capacity for gas exchange from one surface by the other. Addressing these gaps is essential, as developing crops that maintain high photosynthetic performance while exhibiting improved WUE under fluctuating environmental conditions is of great interest, and surface-specific manipulation of stomatal features could represent an exciting approach to potentially develop crops with improved WUE.

In this study, we examined stomatal responses to red and blue light in wheat (*Triticum aestivum*) lines overexpressing *Triticum aestivum* EPIDERMAL PATTERNING FACTOR1 (TaEPF1) (TraesCS2B02G556200), which have reduced SD (Hughes et al. 2017; Dunn et al. 2019). By integrating dynamic gas exchange measurements with detailed anatomical and molecular analyses, we explore how EPF1 overexpression differentially modulates stomatal development and responsiveness when illumination was provided to the adaxial or abaxial surfaces. Our work provides important insights into the compensatory mechanisms, particularly the enhanced blue light-induced stomatal opening that may enable plants with reduced SD to optimize gas exchange and WUE under complex, fluctuating light environments.

## Results

### Overexpression of EPF1 reduces SD and maximum anatomical conductance differently on adaxial and abaxial leaf surfaces

**Figure 1.**
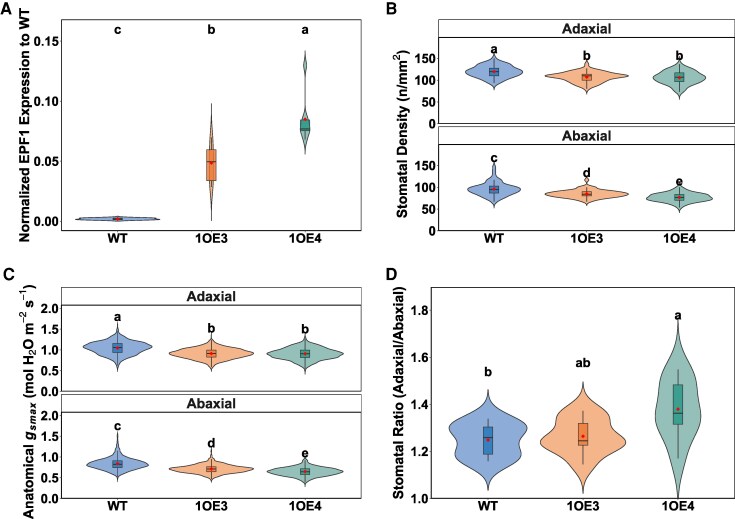
Impact of *EPF1* overexpression on gene expression and stomatal traits in wheat. **A)** EPF1 expression levels in WT (green) and transgenic lines 1OE3 and 1OE4, normalized to WT levels (*n* = 8). **B)** SD measurements from adaxial and abaxial leaf surfaces (*n* = 56). **C)** Calculated maximum anatomical stomatal conductance (*g*_smax_) for both leaf surfaces based on SD and guard cell length (*n* = 56). **D)** Ratio of adaxial to abaxial SD (*n* = 8). Red symbols indicate means. Violin plots display probability density distributions with embedded narrow box plots showing median (center line), interquartile range (box limits), and 1.5× IQR whisker extensions, while red diamonds indicate arithmetic means and outliers are incorporated within the violin contours rather than displayed as separate points. Note: Guard cell length (*n* = 168) is provided in Supplementary material.

TaEPF1 transcript abundance was significantly (*P* < 0.05) elevated in transgenic lines compared with wild-type (WT) (Fig. 1A). This increased *EPF1* expression was tightly associated with reduced SD on both leaf surfaces (*n* = 56). In WT plants, Sds were 119.5 ± 13.2 stomata mm⁻² (adaxial) and 95.8 ± 15.5 mm⁻² (abaxial). The transgenic lines exhibited significantly (*P* < 0.05) lower densities; however, these differed on the 2 surfaces. On the adaxial surface, Sd was significantly (*P* < 0.05) reduced to 107.2 ± 11.3 mm⁻² (1OE3) and 105.5 ± 14.6 mm⁻² (1OE4). Abaxial reductions were more pronounced, with decreases of 11.5% in 1OE3 (84.8 ± 8.9 mm⁻²) and 19.9% in 1OE4 (76.7 ± 9.7 mm⁻²) compared with WT (Fig. 1B). While the reductions in adaxial Sd were similar between the 2 transgenic lines (∼11% to 12%), the higher-expressing 1OE4 line showed a more substantial decrease on the abaxial surface (∼20%), indicating differential effects of EPF1 overexpression between leaf surfaces. Contrary to expectations, stomatal size (measured as guard cell length) was reduced in both transgenic lines compared with WT; again, this decrease was greatest on the abaxial surface (Supplementary Fig. S1). Maximum anatomical stomatal conductance (*g*_smax_) calculated using SD and guard cell dimensions (*n* = 56 per genotype per surface) was significantly (*P* < 0.05) reduced in transgenic lines (Fig. 1C). Adaxial *g*_smax_ decreased by 12.9% (1OE3) and 13.4% (1OE4) compared with WT (*P* < 0.05). Abaxial reductions were more substantial at 14.8% (1OE3) and 22.6% (1OE4) (*P* < 0.05). The adaxial and abaxial SD ratio was affected by *EPF1* overexpression, with WT plants showing a ratio of 1.25 ± 0.0123 and 1OE3 maintaining a similar ratio (1.26 ± 0.0132). However, 1OE4 exhibited a significant 10.4% increase (*P* = 0.0285) to 1.38 ± 0.022 compared with WT.

### Reduced SD alters photosynthetic performance and stomatal responses to blue light

**Figure 2.**
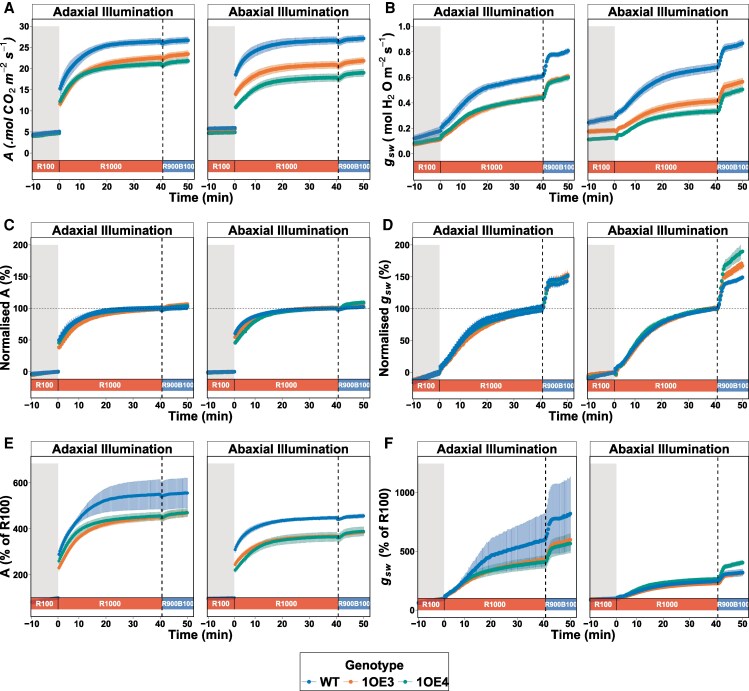
Dynamic responses of photosynthesis rate (*A*) and stomatal conductance (*g*_sw_) to step changes in PPFD and light quality: **A)** time course of photosynthesis rate (*A*) in response to a step increase in light intensity from 100 (R100) to 1000 µmol m^−2^ s^−1^ (R1000) under 100% red light, followed by exposure to a combination of red and blue light (R900B100, 900 µmol m^−2^ s^−1^ red + 100 µmol m^−2^ s^−1^ blue applied to either adaxial and abaxial leaf surfaces for an additional 10 min). **B)** corresponding time course of stomatal conductance (*g*_sw_) under the same light conditions. **C)** and **D)** are the normalised responses of *A* and *g*_sw_, respectively, relative to the differential between the R100 and R1000 steady states, with the dashed horizontal line at 100% indicating the R1000 steady state. The vertical dashed line marks the transition from R1000 to R900B100. Data are presented as mean ± standard error (*n* = 8 to 9) for all genotypes. The gray shaded area in each panel indicates the period of low PPFD (R100) prior to the step increase to R1000.

**Figure 3.**
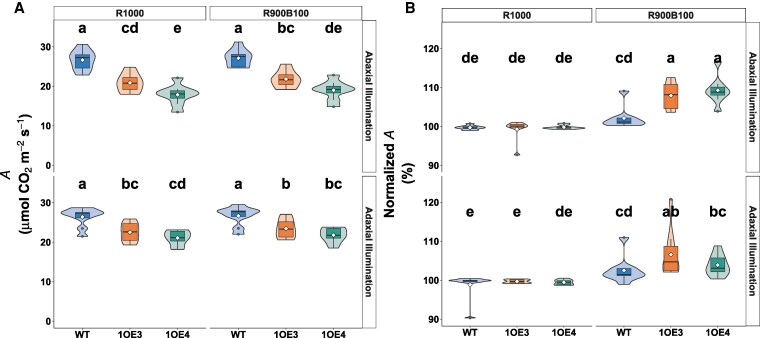
Steady-state net photosynthetic rates (*A*) in WT and EPF1 lines 1OE3 and 1OE4 under different light conditions. **A)** Absolute *A* (μmol CO_2_ m⁻² s⁻¹). **B)** Normalized *A to steady state A* at R100 and R1000, indicating the relative performance under subsequent R900B100 light phase (*n* = 8). Different letters denote statistically significant differences between groups (*P* < 0.05) based on Lsd tests. Violin plots display probability density distributions with embedded narrow box plots showing median (center line), interquartile range (box limits), and 1.5× IQR whisker extensions, while red diamonds indicate arithmetic means and outliers are incorporated within the violin contours rather than displayed as separate points.

Reduced SD in TaEPF1-overexpressing lines (1OE3 and 1OE4) affected photosynthetic performance and stomatal dynamics. Under low PPFD conditions of red-only light (R100), both photosynthetic rate (*A*) and stomatal conductance (*g*_sw_) were significantly (*P* < 0.05) higher in WT compared with transgenic lines, with these differences most pronounced on the abaxial surface and the 1OE4 line, which had the greatest EPF1 expression levels (Fig. 2A and B). Following a step increase in light to 1000 µmol m⁻² s⁻¹ (R1000), *g*_sw_ increased at similar rates across genotypes. However, absolute values of both *A* and *g*_sw_ remained elevated in WT. On the abaxial surface, 1OE3 maintained higher *A* and *g*_sw_ compared with 1OE4 (Fig. 2A and B). The substitution of 10% red light with blue light (R900B100) induced rapid and substantial increases in *g*_sw_ across all lines relative to the steady R1000 phase. The kinetics of this *g*_sw_ response were notably faster than those observed during the initial red-light transition. While WT maintained higher absolute *g*_sw_ values on both surfaces (Fig. 2B), the transgenic lines exhibited larger percentage increases in *g*_sw_, particularly 1OE4 when the abaxial surface was illuminated (Fig. 2D). Photosynthetic rate (*A*), however, remained relatively unchanged in WT following blue light addition (Fig. 2C), meaning that enhanced *g*_sw_ did not translate to increased carbon assimilation and that photosynthesis was not significantly limited by *g*_sw_ under steady-state R1000. However, *A* increased by between 5% and 10% in transgenic lines under abaxial illumination, indicating the removal of a diffusional constraint (Fig. 3).

### Surface-specific enhancement of stomatal blue light responses

**Figure 4.**
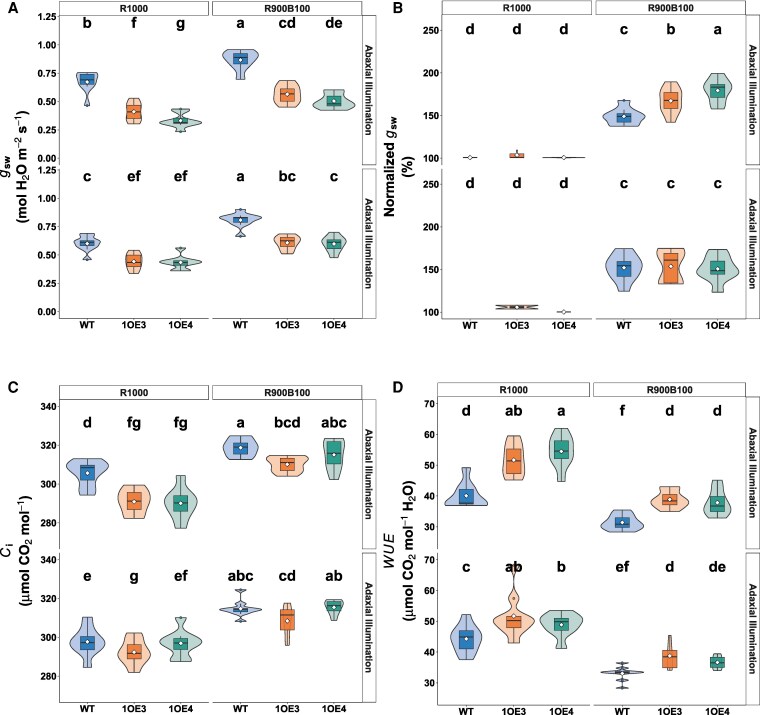
Steady-state gas exchange parameters in WT and EPF1-overexpressing wheat under different light conditions. **A)** Absolute stomatal conductance (*g*_sw_), **B)** stomatal conductance normalized to R1000 values (set as 100%), showing relative increase under R900B100, **C)** intercellular CO_2_ concentration (*C_i_*), and **D)** WUE (calculated as *A*/*g*_sw_). Data are shown for WT, and transgenic lines 1OE3 and 1OE4 (*n* = 8 to 9 per genotype). Different letters indicate statistically significant differences between groups (*P* < 0.05). Different letters indicate statistically significant differences between groups (*P* < 0.05). Violin plots display probability density distributions with embedded narrow box plots showing median (center line), interquartile range (box limits), and 1.5× IQR whisker extensions, while red diamonds indicate arithmetic means and outliers are incorporated within the violin contours rather than displayed as separate points.

Steady-state gas exchange values were derived from the final 10 measurement points of R1000 and R900B100 light phases under both adaxial and abaxial illumination. Stomatal conductance (*g*_sw_) increased substantially in response to blue light (Fig. 4A). Under abaxial illumination, WT plants showed an increase in *g*_sw_ from 0.672 ± 0.096 to 0.867 ± 0.086 mol m⁻² s⁻¹ (*P* < 2 × 10⁻¹⁶). Similar significant (*P* < 0.05) increases were observed in transgenic lines, as 1OE3 increased from 0.410 ± 0.081 to 0.565 ± 0.080 mol m⁻² s⁻¹, while 1OE4 rose from 0.331 ± 0.065 to 0.505 ± 0.067 mol m⁻² s⁻¹. However, no significant enhancements were observed under adaxial illumination across all genotypes. When normalized to R1000 baseline values (100%), the relative *g*_sw_ changes revealed distinct blue light sensitivity patterns across leaf surfaces and genotypes (Fig. 4B). Under abaxial illumination, WT showed a modest increase of 49%, while transgenic lines demonstrated a statistically significant (*P* > 0.0001) enhanced responses, with *g*_sw_ in 1OE3 increased by 67% and 1OE4 showed the strongest response of 90%. Adaxial responses were more moderate and similar across all plants, with increases of ca. 59% (WT), 57% (1OE3), and 53% (1OE4).

Intercellular CO_2_ concentration (*C_i_*) increased following blue light exposure (Fig. 4C). Under abaxial illumination, *C_i_* in 1OE3 rose from 289 ± 12 to 308 ± 8 μmol mol⁻¹ (6.6% increase), while 1OE4 showed an 8.6% increase from 290 to 315 μmol mol⁻¹. WT exhibited a smaller increase from 306 to 319 μmol mol⁻¹ (4.3%). It is worth noting that under steady-state R1000, transgenic lines maintained lower *C_i_* compared with WT, suggesting more constrained CO_2_ influx diffusion under red-only illumination. Additionally, *C_i_* values were consistently lower under adaxial versus abaxial illumination across all genotypes.

WUE under steady-state R1000 was significantly higher in transgenic lines compared with WT (*P* < 0.05). Under abaxial illumination, transgenic lines exhibited 15% to 20% higher WUE than WT (WUE ≈ 39.6). Blue light illumination (R900B100) decreased WUE across all genotypes due to mostly disproportionate increases in *g*_sw_ relative to photosynthetic rate (*A*). WUE decreased by approximately 21% in WT and 18% to 23% in transgenic lines. Notably, under abaxial illumination, transgenic lines maintained higher WUE compared with WT under both light conditions, with 1OE4 maintaining 20% to 25% higher WUE even after blue light stimulation, indicating sustained water use advantages in *EPF1*-overexpressing lines despite typical blue light-induced reductions seen in the WT (Fig. 4D).

### Abaxial surface-specific effects of EPF1 overexpression on dynamic stomatal opening kinetics

**Figure 5.**
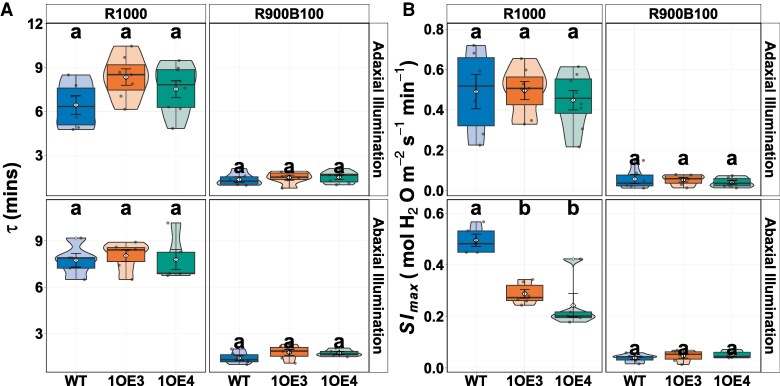
Surface-specific dynamic Stomatal Opening Kinetics in WT, and EPF1-overexpressing lines 1OE3 and 1OE4 under R1000 or R900B100. **A)** shows *τ* (tau), defined as the time to reach 63% of the total increase in stomatal conductance (*g*_sw_), and **B)** depicts Sl_max_, the maximum rate of stomatal opening. Data are presented as violin plots overlaid with boxplots and mean ± SE. Different letters indicate statistically significant differences (*P* < 0.05).

Stomatal opening kinetics were quantified by fitting a sigmoidal model to gas exchange data under the red-only light phase (R1000) and a subsequent mixed red/blue light phase (R900B100). This analysis yielded 2 key parameters: *τ*, the time required to achieve 63% of the total increase in stomatal conductance (*g*_sw_), and Sl_max_, the maximum slope of stomatal opening, which relates the effect on *g*_sw_ to the SD. Under adaxial illumination during R1000, *τ* values were 6.43 ± 1.56 min in WT, 8.34 ± 1.48 min in line 1OE3, and 7.53 ± 1.62 min in line 1OE4 (Fig. 5A). Although the transgenic lines tended to exhibit slightly longer response times, these differences were not statistically significant (*P* > 0.1). Similarly, Sl_max_ under adaxial illumination during R1000 was not statistically different among genotypes. Additionally, no significant differences were observed for either parameter under R900B100 conditions on the adaxial surface (*P* > 0.7). In contrast to adaxial results, under abaxial illumination during the R1000 phase, *τ* values were similar across all genotypes. However, Sl_max_ differed significantly (*P* = 0.000114), with WT plants exhibiting an Sl_max_ of 0.496 ± 0.052 mol H₂O m⁻² s⁻¹ min⁻¹, whereas both transgenic lines showed significantly reduced slopes (0.288 ± 0.041 in 1OE3 and 0.243 ± 0.102 in 1OE4; *P* < 0.05), corresponding to reductions of approximately 42% and 51%, respectively, relative to WT. Under R900B100 on the abaxial surface, neither *τ* nor Sl_max_ differed among genotypes (Fig. 5B). While the overall timing of stomatal opening (*τ*) remains largely unaffected by EPF1 overexpression, the maximum slope of stomatal opening (Sl_max_) is affected in EPF1-overexpressing lines under abaxial illumination during high-intensity red-only light.

### Enhanced stomatal efficiency under blue light compensates for reduced density

**Figure 6.**
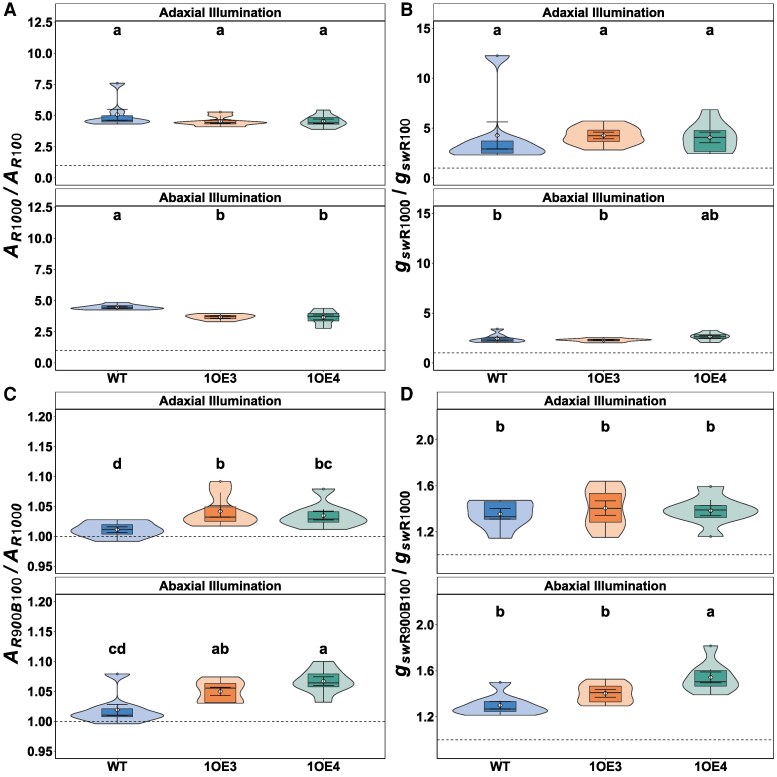
Light response ratios on differential red and blue light sensitivities. **A)** Photosynthetic rate ratio *A_R1000_*/*A_R100_* indicating red light response magnitude. **B)** Stomatal conductance ratio *g_R1000_*/*g_R100_*. **C)** Blue light modulation of photosynthesis (*A_R900B100_*/*A_R1000_*). **D)** Blue light enhancement of stomatal conductance (*g_R900B100_*/*g_R1000_*). Violin plots display probability density distributions with embedded narrow box plots showing median (center line), interquartile range (box limits), and 1.5× IQR whisker extensions, while red diamonds indicate arithmetic means and outliers are incorporated within the violin contours rather than displayed as separate points. Colors represent WT, 1OE3, and 1OE4. Different lowercase letters indicate statistically significant differences (*P* < 0.05) determined by 2-way ANOVA (genotype × surface) followed by Fisher's Lsd post hoc test (*n* = 8 to 9 per genotype).

To further assess whether individual stomata in EPF1-overexpressing lines exhibited altered functional capacity, we normalized *g*_sw_ responses (absolute value change as Δ*g*_sw_) by Sd (Fig. 6). Under red light, stomatal efficiency (Δ*g*_sw_/SD) was significantly (*P* < 0.05) reduced in transgenic lines compared with WT. Adaxial illumination decreased efficiency by 7.4% in 1OE3 and 13.2% in 1OE4 relative to WT (0.180 ± 0.004 mol H₂O m⁻² s⁻¹ mm⁻²). The reduction was more evident when illumination was from the abaxial surface, where transgenic lines showed decreases of 16.4% (1OE3) and 22.0% (1OE4) compared with WT. Remarkably, blue light exposure revealed a dramatic compensatory response in transgenic lines. While WT plants showed modest increases in stomatal efficiency under blue light (0.00195 ± 0.0005 and 0.00438 ± 0.001 mol H₂O m⁻² s⁻¹ mm⁻² for adaxial and abaxial surfaces, respectively), transgenic lines exhibited surprising enhancements, especially under abaxial illumination, with stomatal efficiency increasing 165% in 1OE3 and 237% in 1OE4. The ratio of blue to red light responses within genotypes further highlighted this compensatory mechanism. While WT plants showed blue:red response ratios of 0.011 (adaxial) and 0.020 (abaxial), transgenic lines exhibited substantially higher ratios, reaching 0.087 in 1OE4 under abaxial illumination, which is a 4.3-fold increase compared with WT.

## Discussion

### TaEPF1 overexpression differentially affects SD on leaf surfaces

Our findings support the substantial evidence that TaEPF1 overexpression decreases SD, and the decrease was consistent with increased *EPF1* transcript levels in transgenic lines (Fig. 1A), aligning with earlier studies that have demonstrated EPF1 as key a negative regulator of Sd by inhibiting the formation of stomatal precursors (Hughes et al. 2017; Caine et al. 2019; Dunn et al. 2019). Notably, our study showed in wheat that overexpression of *EPF1* differentially influences Sd on the abaxial and adaxial leaf surfaces, with a more pronounced decrease in Sd on the abaxial surface (Fig. 1B). This differential response suggests either varied EPF1 expression or enhanced activity of inhibitory signal(s) and/or downstream effectors within this epidermal patterning domain. While distinct developmental pathways governing stomatal patterning across leaf surfaces are documented in dicots, including Arabidopsis (Jalakas et al. 2024), less information is available for monocots. Recent research by Jalakas et al. (2024) further supports the notion that stomatal development is at least partly independently regulated in the adaxial and abaxial epidermis, and there is a need to identify key abaxial-specific regulators and promoters (i.e. ICE Transcription factors, IDD16) (Lee et al. 2017; Qi et al. 2019 ; Zoulias et al. 2021). This differential reduction in Sd directly impacts anatomical maximum stomatal conductance (*g*_smax_), which provides a measure of the theoretical *g*_sw_ capacity based on anatomical features and assuming fully open pore apertures (Fig. 1C). Substantial reductions on the abaxial surface in the high expression line 1OE4 (Fig. 1C) demonstrate that reduced Sd fundamentally constrains the maximum potential conductance for gas exchange with potential implications for physiological performance under variable environmental conditions (McElwain et al. 2016; Pflüger et al. 2024).

### Consequence for gas exchange and photosynthetic performance

The reduction in Sd observed in TaEPF1-overexpressing lines manifests directly in diminished *g*_sw_ and gas exchange. The reduced assimilation rate (*A*) and stomatal conductance (*g*_sw_) under red light only condition (R1000), in the transgenic lines compared with WT (Fig. 2A and B), aligned with the well-established relationships between SD, *g*_sw_, and *A* (Franks and Beerling 2009; Lawson and Blatt 2014) and is supported by the strong relationship between *A* and *g*_sw_ observed in both WT and transgenic lines, which demonstrates *A* dependence on *g*_sw_ (Supplementary Fig. S2). The anatomical constraints imposed by reduced Sd and therefore *g*_sw_ on photosynthetic carbon gain are more apparent in the line with the greatest decrease in Sd and when illumination was provided to the abaxial surface (Fig. 2). However, both transgenic lines also showed decreased stomatal sensitivity to red light, and this was particularly evident when abaxial illuminated (Fig. 6), suggesting both anatomical and functional limitations. Irrespective of the direction of illumination in the WT plants, *A* was nearly identical, demonstrating no difference in photosynthetic potential when light is received at either the abaxial or adaxial surface. Therefore, any decrease in *A* observed between the WT and transgenic lines is clearly driven by diffusional constraints as a result of low Sd and reduced stomatal sensitivity, resulting in reduced *g*_sw_, confirming a fundamental constraint for gas exchange capacity (Supplementary Fig. S2). It is well established that low *g*_sw_ can limit CO_2_ uptake for photosynthesis when consumption capacity is high (Wong et al. 1979; Lawson et al. 1998), reducing *C_i_* and therefore photosynthesis (Morison et al. 2007; Morison and Lawson 2007).

Several studies have demonstrated that plants with altered Sd maintain similar *g*_sw_ values to plants with no alterations in density, as changes in pore aperture compensated for any change in density (Bussis et al. 2006; Lunn et al. 2024). Rather than fully compensating for reduced numbers by increasing stomata aperture, the transgenic lines maintain a more conservative *g*_sw_ and reduced sensitivity (Fig. 6). This more conservative stomatal operation resulted in the improved WUE observed in both EPF overexpression lines (Fig. 4D). These findings raise questions regarding the mechanism driving such responses. Previous studies have demonstrated that stomata on the abaxial surface are more responsive to light transmitted through the leaf (Wang et al. 2008), impacting the overall *g*_sw_ response. The fact that much larger differences in *g*_sw_ were observed when illumination was provided from the abaxial surface suggests a mesophyll signal is involved in these responses (Lawson and Blatt 2014). Numerous reports have suggested that mesophyll signal(s) driven by mesophyll photosynthesis control stomatal opening (Lee and Bowling 1992; Lee and Bowling 1995; Messinger et al. 2006; Fujita et al. 2013) and are a key component to the red-light response (Lawson et al. 2018). The importance of this unknown signal has been demonstrated by comparing stomatal responses in epidermal peels to intact material (Fujita et al. 2013), and in floating peels in solutions with illuminating mesophyll cells or chloroplasts (Lee and Bowling 1992; Lee and Bowling 1995), and epidermal–mesophyll transfer experiments (Mott et al. 2008; Fujita et al. 2013; Fujita et al. 2019; Santos et al. 2021). However, the same was not observed in WT plants, suggesting some additional constraints in the EPF lines, driven by possible differences in SD, size, or stomatal sensitivity in the transgenics.

### Enhanced stomatal blue light responsiveness acts as a compensatory mechanism

Perhaps the most significant finding of this study is the reduced stomatal red-light responsiveness and enhanced responsiveness to blue light (R900B100) in EPF1 *overexpressors* particularly under abaxial illumination (Figs 2D and 3B; Fig. 6). Under red light stomata efficiency (Δ*g*_sw_/SD) was greatly reduced in the transgenic lines compared with WT irrespective of surface illumination, although the extent of the decrease was greatest when abaxial illuminated, where the drawn down of *C_i_* was greatest (Fig. 4C), confirming recent studies showing equal *C_i_*-dependent and *C_i_*-independent stomatal responses to red light (Taylor et al. 2024). The larger percentage increase in *g*_sw_ observed in the transgenic lines with blue light was more than double (in 1OE4) that of the increase observed in the WT. This heightened responsiveness also coincides with lower *C_i_* in both transgenic lines under steady-state conditions (Fig. 4C), suggesting that enhanced blue light sensitivity may serve as a compensatory mechanism for photosynthetic limitations imposed by reduced SD.

Our findings align well with earlier observations that stomata on the abaxial epidermis tend to be inherently more light sensitive to specific wavelengths (Travis and Mansfield 1981). However, this sensitivity appears to be species specific, with no difference in sensitivity between abaxial and adaxial stomata reported for *Vicia faba* (Yera et al. 1986) and cotton (Lu et al. 1993). Wang et al. (2008), using a lab-built gas exchange system capable of measuring light responses of 2 leaf surfaces independently, showed that the sensitivity to light, especially blue light, was higher in the abaxial stomata than in the adaxial stomata in sunflower, supporting our observations. It has been proposed that surface-specific sensitivity particularly to blue light is due to a higher concentration of the blue light photoreceptors in abaxial than adaxial guard cells (Goh et al. 1995) and/or that guard cells retain phot1 in the plasma membrane after exposure to blue light more than other cell types (Wan et al. 2007; Wang et al. 2008). Such observations likely underpin the rapid and robust blue light-induced stomatal opening observed here, especially on the abaxial surface, where EPF1 overexpression caused a more pronounced reduction in Sd and hence a greater relative demand for compensatory opening (Büssis et al. 2006). Interestingly, here we show reduced stomatal sensitivity to red light when Sd is lower, contradicting those studies that have suggested function compensates for anatomical changes (Büssis et al. 2006). These findings point toward a complex relationship between Sd and function in such compensatory responses (Fig. 6).

It is well established that there is a close relationship between *A* and *g*_sw_ that is driven by mesophyll demands for CO_2_ (Wong et al. 1979; Xiong et al. 2018; Sakoda et al. 2020), driven by changes in *C_i_* and other unknown *C_i_*-independent signal(s) (Lawson et al. 2014; Santelia and Lawson 2016; Lawson et al. 2018; Taylor et al. 2024). Although this “mesophyll signal” is associated with the red light, and the blue light response is considered independent, several studies have reported direct links between the 2 pathways. HIGH TEMPERATURE 1 (HT1), which is involved in both the red light responses (Matrosova et al. 2015) and CO_2_ signaling in guard cells (Pankasem et al. 2024), has been shown to be involved in activation of the MAP3K Kinase CONVERGENCE OF BLUE LIGHT AND CO2 1 and 2 (CBC1/CBC2) involved in blue light stomatal opening (Takahashi et al. 2022). Hiyama et al. (2017) further showed that CBC1/2 kinases integrate both blue light and CO_2_ signals by inhibiting S-type anion channels, facilitating stomatal opening by integrating signals from blue light and low *C_i_*. The consistently low *C_i_* observed in the EPF1-overexpressing lines under red-only light (R1000) was insufficient to drive sufficient aperture changes to compensate for reduced density due to reduced sensitivity to red light (Fig. 6), when abaxially illuminated, however, subsequent blue-induced stomatal opening was able to compensate for this red light limitation imposed by lower SD and sensitivity (Fig. 6). This supports the integration of blue light and red light signaling in guard cells for regulating stomatal opening, and it is the integration of these signaling responses that determines *g*_sw_. Our observation that the reduction in red light sensitivity and magnitude of blue light response is associated inversely with SD, with the greater differences observed in the 1OE4 lines with the lowest abaxial SD supports an integrated signal.

Our dynamic analysis of stomatal opening kinetics further supports this. While the time required to achieve 63% of maximal conductance (*τ*) remained consistent across genotypes, the maximum increase in stomatal conductance (Sl_max_) was reduced in transgenic lines, particularly during abaxial illumination with red-only light (R1000). This kinetic trade-off reflects the inherent limitation imposed by both a reduced number of stomatal units and reduced sensitivity (Fig. 6). While the speed of stomatal opening (*τ*) is maintained, the overall magnitude of change in conductance is constrained by fewer pores and lower aperture under red light. This is important in fluctuating light environments where both rapid and overall magnitudes of response are critical for maximizing CO_2_ uptake (Shimazaki et al. 2007; Vialet-Chabrand and Lawson 2019). However, the enhanced blue light response observed in the EPF1 *overexpressors* compensates for the reduced opening kinetics under red light, thereby supporting sustained photosynthetic performance. These findings highlight the importance of the stomatal blue light responses for maintaining photosynthetic potential (Matthews et al. 2020) and the complexity and potential co-ordination between stomatal red and blue light responses.

An alternative possible explanation for the differential responses between the 2 surfaces could also be the differences in the [CO_2_] or *C_i_* concentrations at those leaf surfaces. Recently, Askanbayeva et al. (2024) used ^13^C abundance from epicuticular waxes from hypostomatous species to show lower [CO_2_] at the adaxial surface compared with the abaxial surface, whilst in amphistomatous species (such as wheat), [CO_2_] at each surface was dependent on SD. When illumination is directed at the abaxial surface as in our experimental protocol, this could create a particularly strong CO_2_ limitation scenario in EPF1-overexpressing lines due to the substantially reduced abaxial Sd (Fig. 1B) and altered ratio of adaxial to abaxial stomatal distribution (Fig. 1D). The resulting steeper CO_2_ gradient in transgenic lines under abaxial illumination could trigger more robust mesophyll-to-guard cell signaling cascades specifically directly on this surface. Further research is needed to quantify these hypothesized CO_2_ gradients across leaf tissues with altered AD/AB Sd ratios (Márquez et al. 2023).

### Trade-off between carbon assimilation and water conservation

Our findings also contribute substantial insight into the fundamental trade-off between carbon assimilation and water conservation (Fig. 4D). While EPF1 *overexpressors* exhibited reduced assimilation rates due to constrained *g*_sw_ (Fig. 2A and C), these anatomical limitations were accompanied by improvements in WUE. It is remarkable how the transgenic lines achieved a consistent 15% to 20% advantage in WUE compared with WT even following blue light application (Fig. 4D), which is typically associated with reduced WUE due to disproportionate increases in *g*_sw_ (Matthews et al. 2020; Vialet-Chabrand et al. 2021). This trade-off holds particular relevance for drought tolerance mechanisms and illustrates the complexity of interactions between anatomical and functional traits in *g*_sw_ behavior and the potential to exploit these for improved crop performance. Plants exhibiting reduced SD coupled with enhanced WUE likely demonstrate superior adaptation to water-limited environments, effectively minimizing transpirational water loss while maintaining adequate photosynthetic capacity during optimal light periods. Similar WUE enhancements following genetic manipulation of SD have been documented across various crop species, including wheat and rice plants (Franks et al. 2015; Caine et al. 2019; Dunn et al. 2019). Importantly, our work extends these observations to differential surface-specific patterning and impact on responses in wheat under different spectral compositions. While supporting that EPF1 overexpression represents a viable strategy for developing cultivars with enhanced drought resilience, we also show the possibility of developing future drought-resistant or photosynthetically efficient cultivars by manipulating genes, including but not limited to the EPF family, but doing so in a leaf surface-specific manner.

## Materials and methods

### Plant material and growth conditions

*T. aestivum* cv. Fielder transgenic lines overexpressing the *TaEPF1* gene (1OE3 and 1OE4), under the control of the rice actin promoter (Dunn et al. 2019) were germinated under controlled environmental conditions, with a 12-h photoperiod at a stable temperature range of 21 to 22 °C. Nontransformed Fielder plants served as the WT control. Plants were grown under these conditions for 2 wk, after which seedlings were transplanted into 1L pots and grown for an additional 7 wk in a greenhouse at the University of Essex (Colchester, England, UK). The greenhouse conditions were as follows: average temperature of 20.9 °C, relative humidity of 45% to 70%, and a 16-h photoperiod (0600 to 2200 h). Natural light was supplemented with high-pressure sodium lamps (HSE NXT 2 600W, HORTILUX SCHRÉDER, the Netherlands) to maintain a minimum intensity of 400 µmol m⁻² s⁻¹ PPFD at the canopy level throughout the photoperiod, as measured with a SpectraPen (PSI, Czech Republic). The average daily light integral was approximately 16.668 mol m⁻² d⁻¹, with light intensities reaching up to 806 µmol m⁻² s⁻¹ PPFD on clear days. Measurements were taken on flag leaves between Zadoks growth stage 45 (boots swollen) to stage 59 (ear emergence complete) (Zadoks et al. 1974). Plants were grown using a randomized complete block design with genotypes randomly distributed across the greenhouse bench. Plants were regularly rotated within the greenhouse bench to minimize positional effects.

### Leaf gas exchange measurements and dynamic model fitting of stomatal kinetics

Leaf gas exchange parameters were measured using Li-6800 infrared gas analyzers with the 6800-01A fluorometer head (Licor Biosciences, NE, USA). The leaf chambers were set to maintain a CO_2_ concentration of 400 μmol mol^−1^, a flow rate of 500 μmol m^−2^ s^−1^, a leaf temperature of 23 °C, and a VPD of 1.2 kPa. To determine responses under varying illumination and across different leaf surfaces, measurements were conducted using bi-directional illumination, illuminating either the adaxial or abaxial leaf surface. Measurements were conducted in a randomized order across multiple days to minimize systemic biases.

To measure assimilation rate (*A*) and stomatal conductance (*g*_sw_), the flag leaf of the wheat plant was positioned in the 6 cm^2^ chamber. Leaf area was determined by measuring leaf blade width at multiple points along the chamber length and calculating the effective area (average width × 3 cm). Each leaf surface was initially acclimated to an intensity of 100 μmol m^−2^ s^−1^ of red light (R100). Leaf gas exchange data were logged at 10-s intervals. After 10 min, the light intensity was increased to 1000 μmol m^−2^ s^−1^ of red light (R1000) and maintained for 30 min. This was followed by a modification in the light spectrum where 10% of the red light was substituted with blue light (475 nm), resulting in a combination of 100 μmol m^−2^ s^−1^ of blue light and 900 μmol m^−2^ s^−1^ of red light (R900B100), which was maintained for a further 10 min. Throughout these light phases, leaf temperature was maintained at ∼23 °C. WUE was calculated as the ratio of assimilation rate (*A*) to stomatal conductance (*g*_sw_) (Supplementary Data Set 1).

The dynamics of stomatal opening in response to the step increase in light intensity (R100 to R1000) and subsequent change in light quality (R1000 to R900B100) were quantified by fitting a sigmoidal function to the temporal *g*_sw_ data, as described in McAusland et al. (2016):


$$g_{s}=(g_{smax}-g_{smin})e^{-e^{\left(\frac{\lambda -t}{k}+1\right)}}+g_{smin}$$

where $\left(g_{smin}\right)$ and $\left(g_{smax}\right)$ represent the minimum and maximum steady state *g*_sw_ value under R100 and R1000 and R900B100 phases, *λ* is the initial response lag time, and *k* is the time constant of stomatal opening. The model was fitted using nonlinear least square regression, and was further validated using a stringent root mean square error threshold of 0.1 for both light phases. Kinetic parameters were then derived from the fitted model, as the time to reach 63% of the total conductance (τ), and the maximum rate of conductance increase (Sl_max_).

### Leaf epidermal impressions and measurements

Surface impressions of the abaxial and adaxial leaf surfaces, excluding the mid-vein, were made using activated polysiloxane impression material (Xantopren L blue, Kulzer). After polysiloxane solidification, impressions were coated with clear nail varnish and transferred onto microscope slides for analysis. SD and guard cell lengths were quantified using a Leica ATC2000 optical microscope with a SWIFT SC500 digital camera. ImageJ software was used to quantify SD and guard cell dimensions from the captured images.

### Estimation of maximum anatomical stomatal conductance

The maximum anatomical stomatal conductance to water vapor $\left(g_{smax}\right)$ was calculated independently for adaxial and abaxial leaf surfaces across all genotypes using a modified equation from Franks and Farquhar (2007) adapted for dumbbell-shaped stomatal morphology characteristic of grasses following (Parlange and Waggoner 1970; Nunes et al. 2022).


$$g_{smax}=\frac{\frac{d_{w}}{v}\times SD\times 0.05148\times GCL^{2}}{0.25\times GCL+0.128\times GCL}$$

where *d_w_* is the diffusivity of water vapor in air at 23 °C (gas exchange temperature), taken as 2.42 × 10⁻⁵ (m² s⁻¹). v is the molar volume of air at 23 °C (0.024 m^3^ mol^−1^). Sd is the stomatal density and GCL is the guard cell length (μm). The coefficients 0.05148 and 0.128 are the grass-specific pore geometry, where pore length equals 0.44GCL and pore width equals 0.13GCL, with a 0.9 correction factor for the hexagonal pore shape, and pore depth approximates 0.25GCL based on the guard cell width at the stomatal center in the dumbbell-shaped complexes (Nunes et al. 2022).

### TaEPF1 expression analysis

Three leaf discs (diameter of 10 mm) were sampled from the flag leaf of glasshouse-grown plants. To ensure RNA integrity, samples were immediately frozen in liquid nitrogen. The frozen leaf tissues were mechanically homogenized using a Qiagen TissueLyser II, and total RNA was isolated from the resultant tissue powder using the NucleoSpin RNA/Protein Kit (Macherey-Nagel), following the manufacturer's instructions. Potential genomic DNA contamination was eliminated using DNase I treatment, followed by purification with the RNA Clean & Concentrator-5 kit (Zymo Research). RNA quantity and quality were assessed using a NanoDrop One spectrophotometer (Thermo Scientific).

For cDNA synthesis, 1 µg of purified total RNA was combined with oligo(dT)12-18 primer (Invitrogen) and the RevertAid Reverse Transcriptase kit (Thermo Scientific), following the manufacturer's guidelines. The synthesized cDNA was subsequently diluted 10-fold. Quantitative PCR (qPCR) was conducted on a Bio-Rad CFX384 Real-Time System using 2× qPCRBIO SyGreen Mix Lo-ROX (PCR Biosystems Ltd). Each 10 µl reaction mixture contained 5 µl of SyGreen mix, 3 µl of the diluted cDNA template, and 0.5 mm each of the forward and reverse primers. The thermal cycling program comprised an initial denaturation at 95 °C for 2 min, followed by 40 cycles of denaturation at 95 °C for 10 s and annealing/extension at 68 °C for 15 s.

Gene expression was normalized using the reference gene *Ta2776* (RNase l inhibitor-like protein) (Paolacci et al. 2009). The relative expression of the *TaEPF1* gene was calculated using the 2−ΔΔCt method. All qPCRs were performed in triplicate to ensure technical reproducibility. The primer sequences used for the amplification were as follows:

*Ta2776* Forward: 5′-CGATTCAGAGCAGCGTATTGTTG-3′

*Ta2776* Reverse: 5′-AGTTGGTCGGGTCTCTTCTAAATG-3′

*TaEPF1* Forward: 5′-ACCGTCCCATCAGGAGAAGCGG-3′

*TaEPF1* Reverse: 5′-CGCACCGGAAGCTGACCATGAC-3′.

### Statistical analysis

Statistical analysis was conducted using R (version 4.3.0). The Shapiro–Wilk test was used for normality. All gas exchange parameters along with their derived metrics (normalized *A*, normalized *g*_sw_, WUE) were analyzed through a multifactorial analysis of variance (ANOVA) to assess genotype effects (WT, 1OE3, and 1OE4), illumination conditions (adaxial/abaxial), and light phase interactions (R1000 and R900B100). Post hoc evaluations used the least significant difference (LSD) test with significance groups (*α* = 0.05) denoted by common letters. Anatomical traits and stomatal kinetic parameters derived from nonlinear modelling (*τ*, Sl_max_) underwent a similar ANOVA assessment with subsequent LSD testing. All statistical tests maintained *α* = 0.05 significance threshold with 2-tailed distributions. Results are presented as means ± standard error or, where appropriate, as median with interquartile range.

To determine if differences in Sd were driven by genetic or environmental parameters, a variance component analysis was used to partition phenotypic variance into genetic (*η*²) and residual environmental components for each trait (SD: *N* = 56 plants per genotype, and all gas exchange derived traits: *N* = 8). Genotype explained 18.8% to 74.0% of the total variance (*η*² = 0.188 to 0.740), which exceeded Cohen's threshold for a large effect (*η*² ≥ 0.14) in every case according to conventional standards (Cohen 2013; Lakens 2013). Physiological traits showed the greatest genetic control (*η*² ≥ 0.590 for all gas exchange variables) (Supplementary Table S1). Measurement precision was high, as within-genotype coefficients of variation averaged 12.0% (range 8.4% to 21.3%) and with comparable CV ranges for the WT and both OE lines (Supplementary Table S1).

### Accession numbers

Sequence data from this article can be found in the GenBank/EMBL data libraries under accession numbers: TaEPF1 (TraesCS2B02G556200). Arabidopsis homologs referenced EPF1 (AT2G20875). Additional wheat gene accessions can be found in the Ensembl Plants database (https://plants.ensembl.org/Triticum_aestivum).

## Supplementary Material

kiaf379_Supplementary_Data

## Data Availability

All primary data are available on request.

## Supplementary data

**Supplementary Figure S1.** Impact of EPF1 overexpression on guard cell length of 2 different leaf surfaces.

**Supplementary Figure S2.** Relationship between net assimilation rate (*A*, μmol CO_2_ m⁻² s⁻¹) and stomatal conductance (*g*_sw_, mol H₂O m⁻² s⁻¹) across different light phases (R1000 and R900B100) and leaf surfaces (adaxial and abaxial).

**Supplementary Figure S3.** Dynamic stomatal opening kinetics under R1000 and R900B100 from WT.

**Supplementary Table S1.** Genotypic contribution to phenotypic variance in stomatal and gas exchange traits.

**Supplementary Data Set S1.** Raw gas exchange, qPCR, Sd and stomatal size data.

## Dive Curated Terms

The following phenotypic, genotypic, and functional terms are of significance to the work described in this paper:

PHOT1 Gramene: AT3G45780
PHOT1 Araport: AT3G45780

PHOT2 Gramene: AT5G58140
PHOT2 Araport: AT5G58140
