# Supplementary material for: Reduced stomatal density in wheat overexpressing EPIDERMAL PATTERNING FACTOR1 differentially affects red and blue light responses
Source: Plant Physiol. 2025 Sep 5;199(1):kiaf379. doi: 10.1093/plphys/kiaf379 (PMC12451101; doi:10.1093/plphys/kiaf379)
Supplement: kiaf379_Supplementary_Data [file kiaf379_supplementary_data.zip › Fan et al _suppl.pdf]

Supplementary Figures

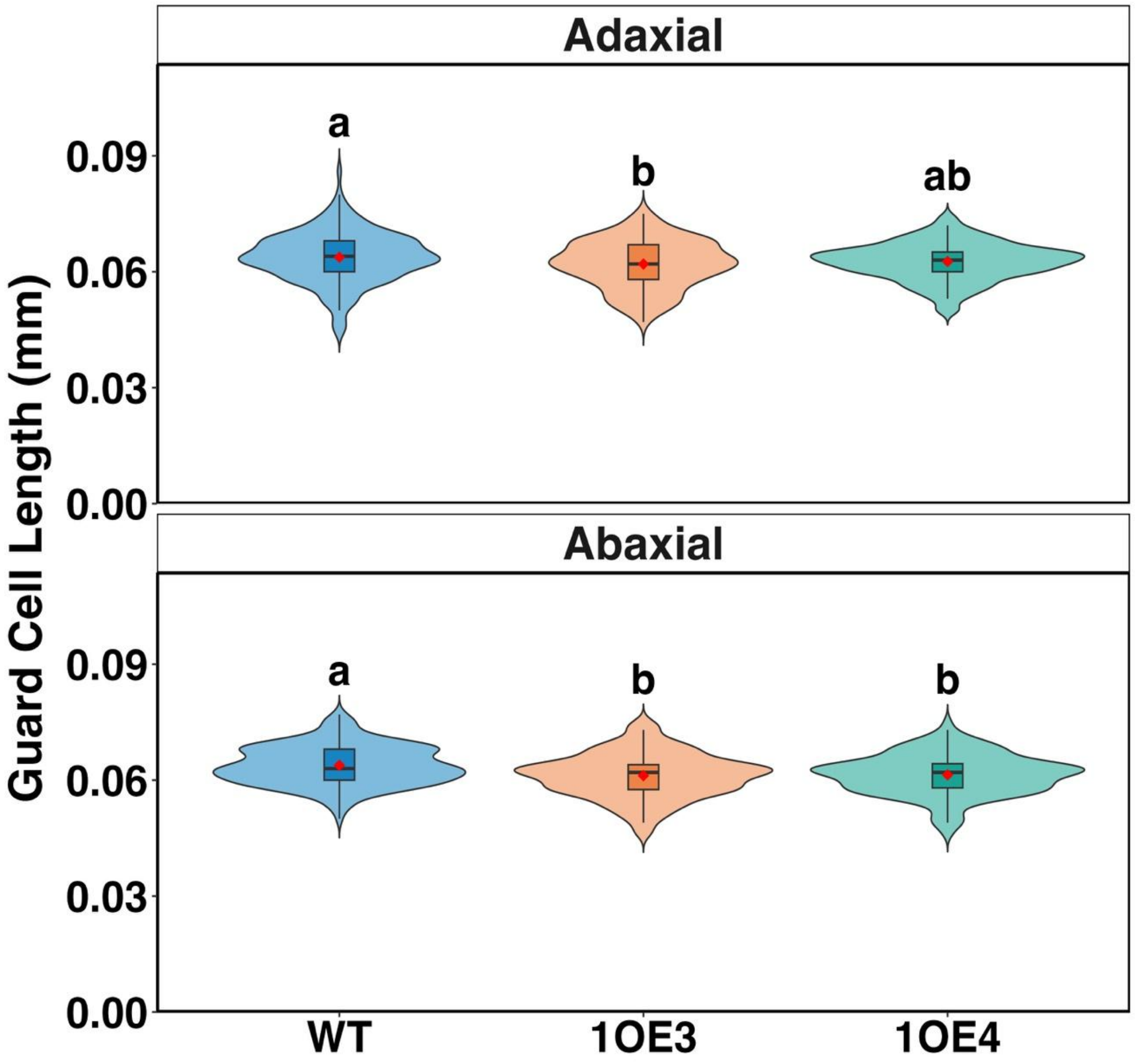

Supplementary Figure S1 Impact of EPF1 overexpression on guard cells length of two different leaf surfaces (n = 168) in WT (blue), 1OE3 (orange), and 1OE4 (teal).

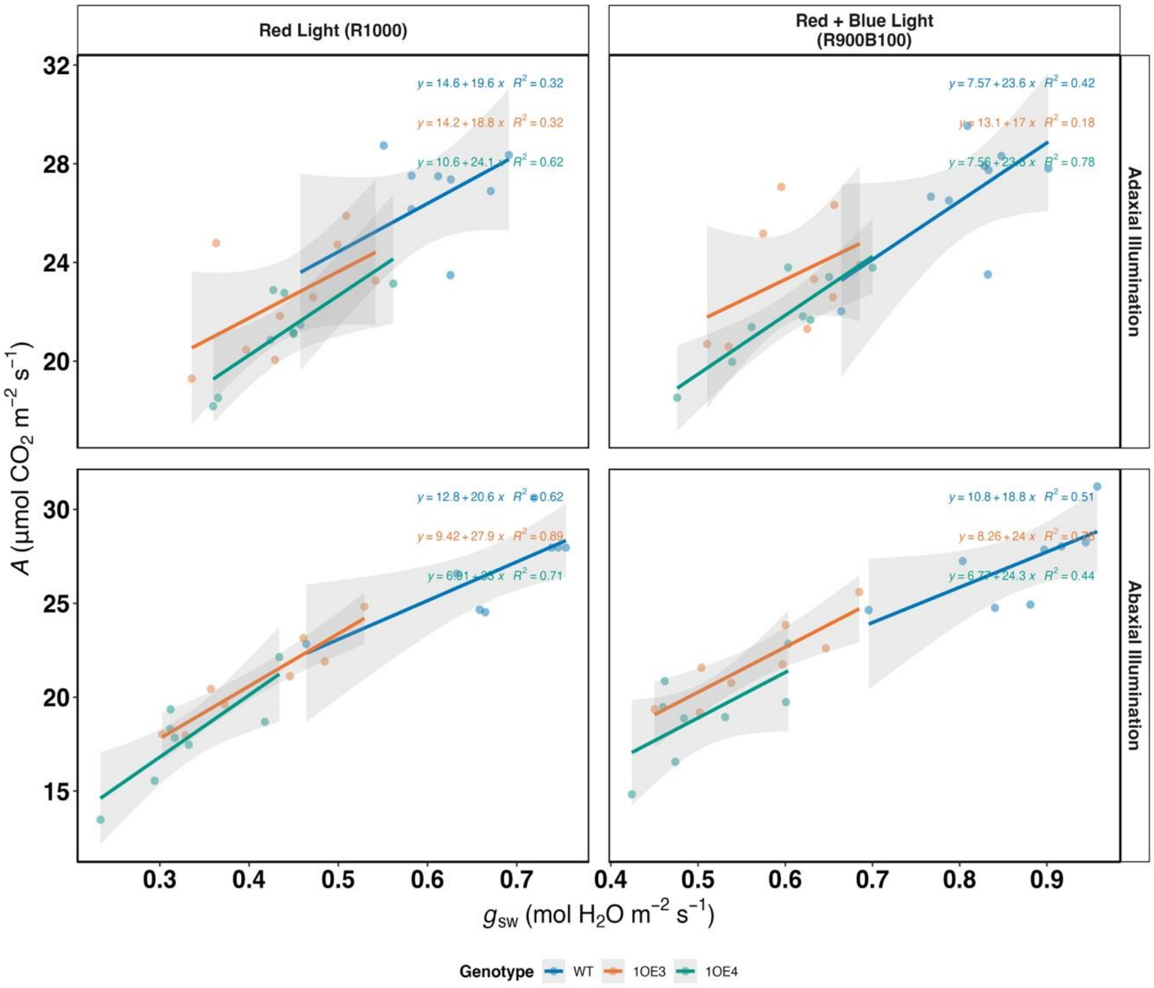

Supplementary Figure S2 Relationship between net assimilation rate (A, µmol CO_2 m^{-2} s^{-1}) and stomatal conductance (g_{sw}, mol H_2O m^{-2} s^{-1}) across different light phases (R1000 and R900B100) and leaf surfaces (adaxial and abaxial) of WT (blue), 1OE3 (orange), and 1OE4 (teal) (n = 8-9 per genotype). Fitted linear regression lines for each line are overlaid with regression equations with R^2 values.

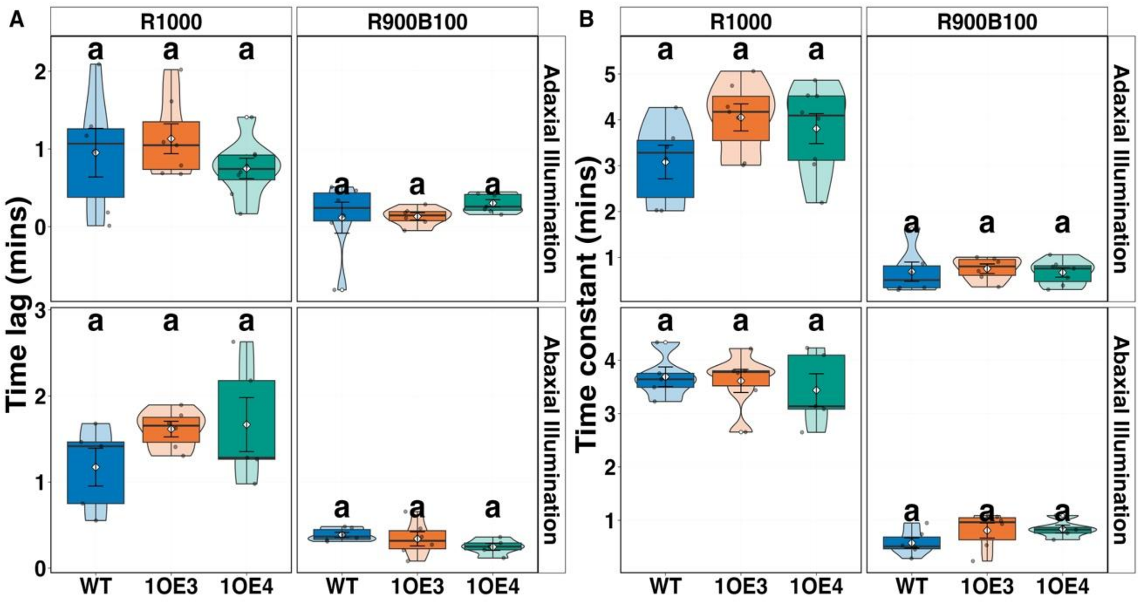

Supplementary Figure S3 Dynamic stomatal opening kinetics under R1000 and R900B100 from WT (blue), 1OE3 (orange), and 1OE4 (teal). (A) shows the initial time lag in minutes after the change in light intensity and spectral composition, and (B) time constant for stomatal opening (*k*) derived from fitting the sigmoidal model. (n = 8-9 per genotype).

Supplementary Table S1. Genotypic contribution to phenotypic variance in stomatal and gas exchange traits

Parameter	N	F	p	η²	Power	CV (WT)	CV (1OE3)	CV (1OE4)
SD (Abaxial)	56	790.1	<0.001	0.3	>0.999	16.1	10.4	12.6
SD (Adaxial)	56	407.8	<0.001	0.2	>0.999	10.9	10.5	13.7
g_{sw} (R1000)	8	45.1	<0.001	0.7	>0.999	13.9	17.4	21.3
g_{sw}(R900B100)	8	67.0	<0.001	0.7	>0.999	9.5	12.0	14.8
A (R1000)	8	33.8	<0.001	0.6	>0.999	9.0	11.2	14.1
A (R900B100)	8	31.0	<0.001	0.6	>0.999	8.5	10.4	12.5

*Variance components analysis partitioned total phenotypic variance into genetic (η²) and residual environmental components. Genetic effect sizes were interpreted following Cohen (2013); (Lakens, 2013) where η² ≥ 0.14 denotes a large effect.

Cohen J (2013) Statistical power analysis for the behavioral sciences. routledge

Lakens D (2013) Calculating and reporting effect sizes to facilitate cumulative science: a practical primer for t-tests and ANOVAs. Frontiers in psychology **4:** 863
